# Bathing in Fevers

**Published:** 1901-06-22

**Authors:** 


### BATHING IN FEVERS.

Many different opinions have been held as to the manner in which baths produce the good effects which undoubtedly do in some cases result from their employment in the treatment of fevers. Some are inclined to hold that it is not merely by abstracting heat that they do good, and even that such lowering of the temperature as follows their application may be due to a modification of heat production quite as much as from an increase of heat loss. It may be said at once then that the experience gained from the use of baths in febrile complaints cannot properly be regarded as proving the utility of the various substitutes for the tub which have been from time to time proposed. So far as the mere abstraction of heat is concerned, however, it is pretty obvious to anyone who has taken part in the performance that to lift a patient out of bed and put him into a bath which has been brought to his bedside, or worse still, to take a patient to a bathroom, as we believe is done in some hospitals where bathing is largely used, is far from being the best possible arrangement for the purpose. In the *Journal of the American Medical Association*, Dr. F. H. Williams has described a method of reducing temperature in fever by the use of what he speaks of as "evaporation baths" which may be found useful in many cases in which the ordinary bath is not available. He places a waterproof under the patient —a folded blanket will do in case of necessity—and then he covers the major part of the trunk and extremities with a single thickness of coarse gauze. This is then sprinkled with water at a temperature of 110° F., and while it is so kept wet the patient is fanned. At first about a pint of water will be sufficient for a single bath, but afterwards more may be used. The rate of evaporation will of course depend upon the temperature and moisture of the surrounding air and upon the amount of fanning which is employed to aid evaporation. It is evident from the careful records taken by Dr. Williams of the temperature, during and after the administration of these baths, that the method is a very useful one, and from the manner in which the descent of the temperature was found to continue for some time after the administration of each bath, their action would seem to approximate very nearly to that of the ordinary so-called tub bath, and certainly they would seem to avoid the over-excitation which is in some cases produced by sponging, which is the method most commonly employed as a substitute when full baths cannot be obtained. Moreover, there is far less slop.

